# GLUT2 expression by glial fibrillary acidic protein-positive tanycytes is required for promoting feeding-response to fasting

**DOI:** 10.1038/s41598-022-22489-2

**Published:** 2022-10-21

**Authors:** M. J. Barahona, F. Langlet, G. Labouèbe, S. Croizier, A. Picard, Bernard Thorens, María A. García-Robles

**Affiliations:** 1 Laboratorio de Biología Celular, Departamento de Biología Celular, Facultad de Ciencias Biológicas, Universidad de Concepción, Concepción, Chile; 2 Center for Integrative Genomics, Faculty of Biology and Medicine, University of Lausanne, Lausanne, Switzerland; 3 Instituto de Neurociencias, Centro Interdisciplinario de Neurociencias de Valparaíso, Universidad de Valparaíso, Valparaiso, Chile; 4 Present Address: Laboratorio de Neurobiología y células madres (NeuroCellT), Departamento de Biología Celular, Facultad de Ciencias Biológicas, Universidad de Concepción, Concepción, Chile; 5 Present Address: Department of Biomedical Sciences, Faculty of Biology and Medicine, University of Lausanne, Lausanne, Switzerland

**Keywords:** Protein transport, Carbohydrates, Histocytochemistry, Neurochemistry, Biochemistry, Cell biology

## Abstract

Feeding behavior is a complex process that depends on the ability of the brain to integrate hormonal and nutritional signals, such as glucose. One glucosensing mechanism relies on the glucose transporter 2 (GLUT2) in the hypothalamus, especially in radial glia-like cells called tanycytes. Here, we analyzed whether a GLUT2-dependent glucosensing mechanism is required for the normal regulation of feeding behavior in GFAP-positive tanycytes. Genetic inactivation of *Glut2* in GFAP*-*expressing tanycytes was performed using Cre/Lox technology*.* The efficiency of GFAP-tanycyte targeting was analyzed in the anteroposterior and dorsoventral axes by evaluating GFP fluorescence. Feeding behavior, hormonal levels, neuronal activity using c-Fos, and neuropeptide expression were also analyzed in the fasting-to-refeeding transition. In basal conditions, *Glut2*-inactivated mice had normal food intake and meal patterns. Implementation of a preceeding fasting period led to decreased total food intake and a delay in meal initiation during refeeding. Additionally, *Glut2* inactivation increased the number of c-Fos-positive cells in the ventromedial nucleus in response to fasting and a deregulation of *Pomc* expression in the fasting-to-refeeding transition. Thus, a GLUT2-dependent glucose-sensing mechanism in GFAP-tanycytes is required to control food consumption and promote meal initiation after a fasting period.

## Introduction

The brain controls energy homeostasis in part through nutrient sensing^1^. Current evidence suggests that a glucose transporter 2 (GLUT2)-dependent glucosensing mechanism controls thermoregulation, glucose homeostasis, and feeding^2–6^. GLUT2, a transporter that belongs to the family of facilitative glucose transporters (GLUTs), is the only known member with low affinity for glucose and high transport capacity (km ∼ 17 mmol/l)^7,8^.

Physiological studies carried out in mice with an inactive *Glut2* gene in the central nervous system (CNS) suggest that GLUT2 is necessary for controlling the autonomic nervous signaling, the first phase of insulin secretion, and pancreatic β-cells during the early postnatal development^9^. A key advance in understanding the GLUT2-dependent brain glucosensing mechanism was development of a genetic reporter system^5,6^. Expression of fluorescent reporter proteins under the control of the GLUT2 promoter together with detailed immunohistochemical analysis showed the localization of GLUT2 in brain regions that control energy homeostasis, such as the posterior brainstem, paraventricular thalamus, and hypothalamus^5,6,10–13^. In the hypothalamus, the expression of GLUT2 was reported in specialized radial glial-like cells, tanycytes, which form the lateral walls and floor of the third ventricle (3V)^10,14,15^. The apical regions of tanycytes directly contacts the cerebrospinal fluid (CSF), and their long basal processes reach different hypothalamic nuclei involved in feeding control^4,16^. Tanycytes have been classified into four subpopulations according to their 3V dorsoventral location and their basal process projections^17,18^. β1–2 tanycytes form the 3V into the infundibulum zone and median eminence (ME), respectively^17,19^. α1–2 tanycytes are located more dorsally in the 3 V; according to their dorsoventral localization, they project laterally towards different neuronal nuclei adjacent to the 3V, such as arcuate nucleus (ARC), ventromedial (VMN), and dorsomedial (DMN)^18^.

We have previously demonstrated that α and β-tanycytes express GLUT2 in their apical region, contacting the CSF^10^. It is essential to highlight that tanycytes can respond to glucose, generating intracellular calcium increases in vitro and ex vivo^14,20^. Whereas it is dependent on glycolysis in β-tanycytes, calcium responses were also evocated by non-metabolizable analogs in α-tanycytes, indicating that both subpopulations could contribute differently to the glucosensing mechanism^14,21^.

The silencing of GLUT2 in ependymocytes and α/β tanycytes in rats decreases satiety, which leads to a rise in body weight^4^. Although neurons were not transduced, deregulation in the expression of orexigenic and anorexigenic hypothalamic neuropeptides was detected in response to glucose injection into the 3V^4^. Thus, GLUT2 inhibition in α and β tanycytes has broad repercussions on feeding behavior.

To examine the role of GLUT2 expression in a specific subpopulation of tanycytes, we inactivated the *Glut2* gene in GFAP-positive tanycytes. Using a spatial distribution analysis of the GFAP-positive tanycytes, we first determined that they are primarily located in the anterior hypothalamic region facing nuclei that control feeding. In animals with in vivo genetic inactivation of the *Glut2* gene, GFAP-expressing tanycytes exhibit decreased total food intake and delayed meal initiation following a fasting period. Unexpectedly, the loss of *Glut2* in GFAP-positive tanycytes led to increased ghrelin plasma concentration during fasting. Additionally, these mice presented an increase in c-Fos expression in the VMN and deregulation of the *Pomc* expression in the fasting-to-refeeding transition. Thus, a GLUT2-dependent mechanism is required in GFAP-positive tanycytes to regulate food intake and establish meal initiation in response to fasting.

## Results

### Both α and β tanycytes express GLUT2 in adult mice

**Figure 1:**
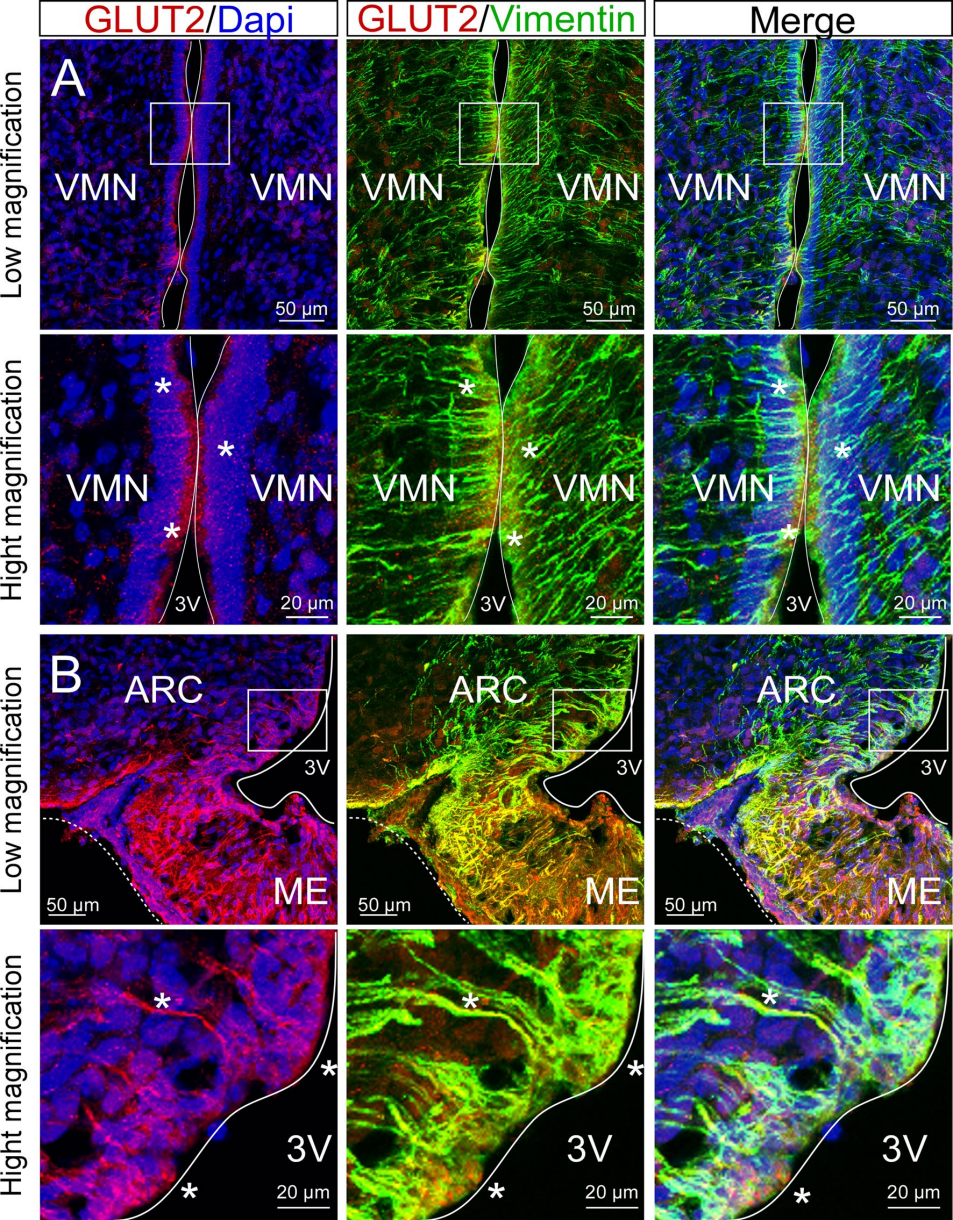
GLUT2 is expressed in hypothalamic tanycytes. Low and high magnification images of tanycytes facing the VMN (**A**) and ARC (**B**) using the anti-GLUT2 (red) and anti-vimentin (green) antibodies. DAPI was used as a nuclear marker. *3V* third ventricle, *ME* median eminence, *VMN* ventromedial nucleus, *ARC* arcuate nucleus.

**Figure 2:**
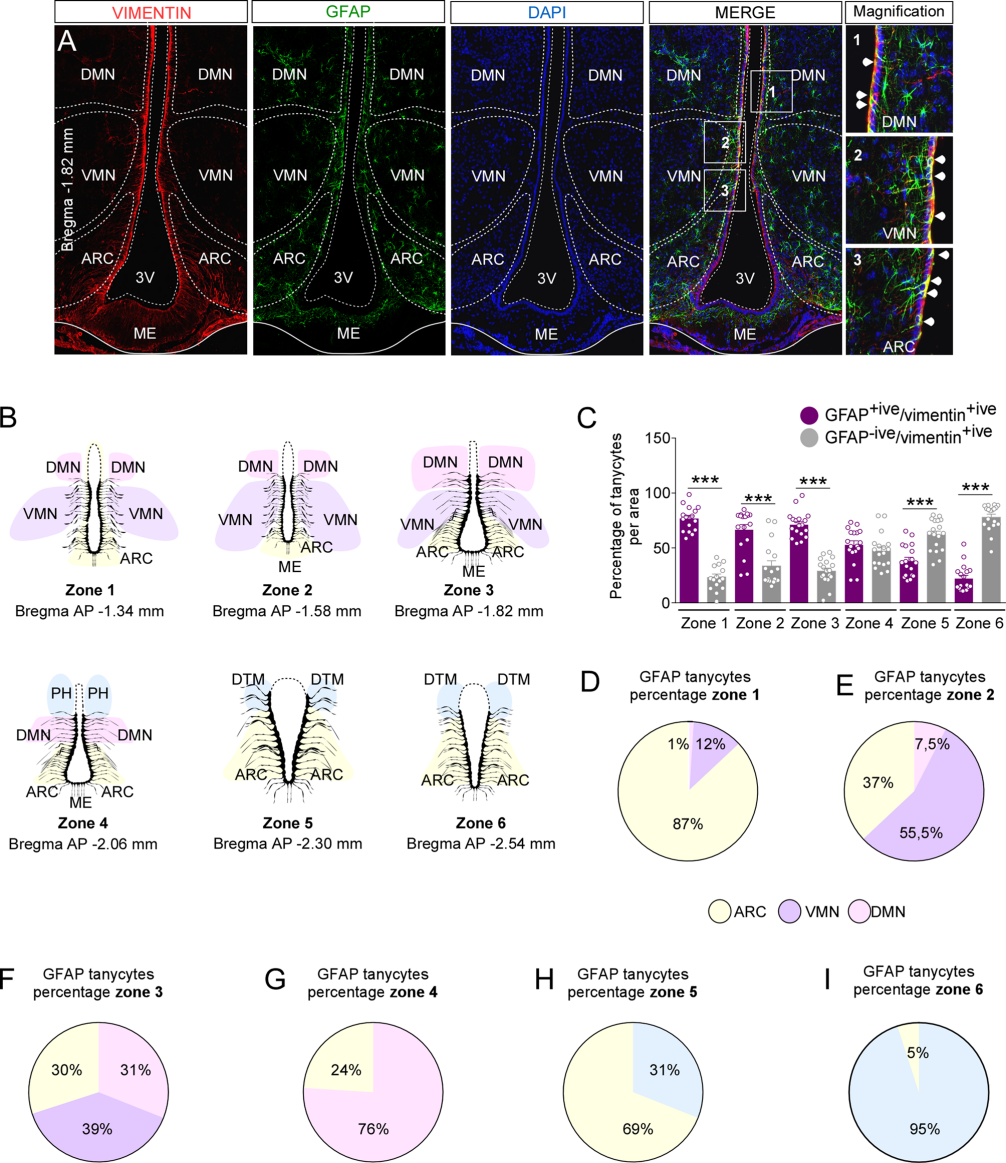
Hypothalamic anteroposterior and dorsoventral distribution of GFAP-expressing tanycytes. (**A**) Coronal sections of the hypothalamic region using the anti-vimentin (red), anti-GFAP (green) antibodies, and the nuclear marker, DAPI (blue). (**B**) Schematic representation of the distribution of anteroposterior tanycytes. (**C**) Percentage of GFAP-positive/vimentin-positive (purpure bars) and GFAP-negative/vimentin-positive (grey bars) tanycytes in the hypothalamic anteroposterior axis (n = 6 mice). (**D**–**I**) Percentage of GFAP-positive/vimentin-positive tanycyte in contact with hypothalamic nuclei in the anteroposterior axis. Four coronal sections were analyzed bilaterally per bregma area. Error bars represent SEM. Multiple comparisons were performed using a one-way ANOVA (Bonferonni’s post-hoc test). ***p < 0.0001.

Before inactivating *Glut2,* its protein localization was evaluated in hypothalamic tissue from bregma AP − 1.82 mm of adult mice. GLUT2 was detected in the cell bodies that form the 3V at the height of VMN (Fig. 1A, asterisk) and the ARC (Fig. 1B). Colocalization with vimentin, a tanycyte marker, was detected in proximal α and β-tanycytes, and a strong reaction was detected in the processes of β-tanycytes that project to the ARC and ME (Fig. 1B, asterisk). Several studies have shown that GFAP-positive tanycytes are mainly located in the VMN and DMN^16,22,23^. At the bregma − 1.82, we observed GFAP-positive tanycytes facing the DMN and VMN that are also vimentin-positive (Fig. 2A). High magnification of the dorsal periventricular regions showed intense colocalization between both intermedia filaments (Fig. 2A,1–3). In contrast, tanycytes facing the ARC and ME are vimentin-positive and GFAP-negative (Fig. 2A). A previous study showed that these tanycytes are GLUT2-positive^4^, and 3D reconstruction confirms GLUT2 immunoreaction and localization in dorsal tanycytes (Supplementary Fig. 1A, arrowheads), which is more evident at high magnification (Supplementary Fig. 1B, arrowheads). In addition, GLUT2 was not detected in parenchymal astrocytes (Supplementary Fig. 1C, arrowhead) as reported previously^10,15^.

Because GFAP-positive tanycytes have been poorly described, we analyzed their anteroposterior location based on 3D GFAP and vimentin immunoreactivity. We evaluated the proportion of GFAP-positive versus GFAP-negative tanycytes on the anteroposterior axis of the hypothalamic areas located between the bregma − 1.34 to the − 2.54 mm. The different bregma locations were identified as zones, as shown in Fig. 2B. Our results showed that GFAP-positive tanycytes are mostly located from zone 1 to 3 (Fig. 2C). In zone 4, we found a transition zone where the percentage of GFAP-positive and GFAP-negative tanycytes is similar. Interestingly, we detected a higher percentage of GFAP-negative tanycytes in zones 5 and 6 of the hypothalamus (Fig. 2C). These observations indicate that GFAP-expressing tanycytes have an asymmetric location along the anteroposterior axis with a marked localization in the more anterior zones. Supplementary Fig. 2 shows the projections of GFAP-positive tanycytes to the different hypothalamic nuclei in detail. Next, we analyzed the hypothalamic dorsoventral location of the GFAP-positive tanycytes from zone 1 to 6. In the anterior region (zones 1 and 2), GFAP-positive tanycytes are mainly located in the VMN and ARC (Fig. 2D,E and Supplementary Fig. 2). Interestingly, in the medial region (zone 3), GFAP-positive tanycytes project into the DMN, VMN, and ARC in similar proportion (Fig. 2F). In the posterior zones of the hypothalamus, GFAP-positive tanycytes contact the DMN, ARC, and the dorsal tuberomammillary nucleus (DTM) (Fig. 2G–I and Supplementary Fig. 2).

### *Glut2* genetic inactivation in GFAP-positive tanycytes

**Figure 3:**
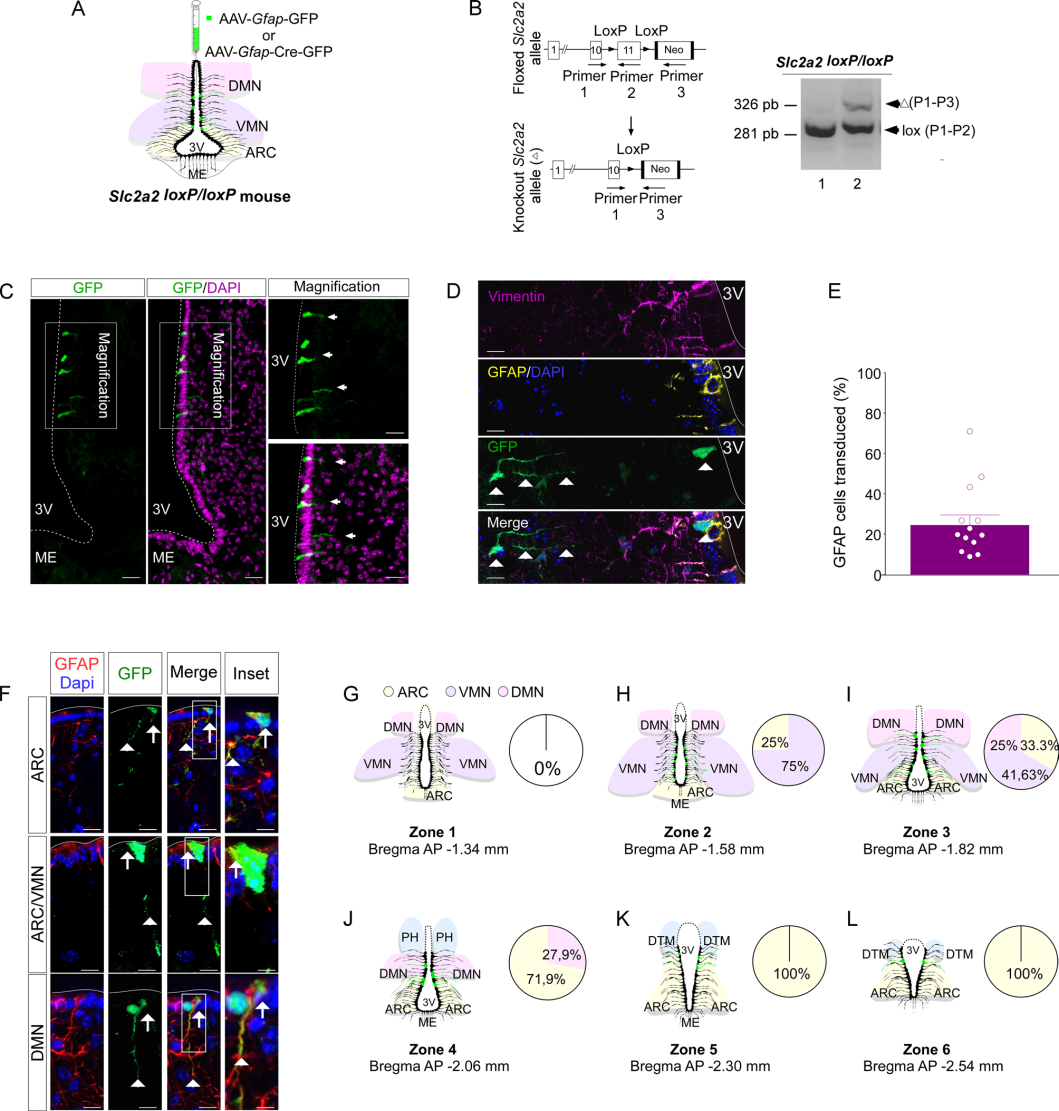
*Glut2* gene inactivation in GFAP-expressing tanycytes and its in situ evaluation. (**A**) Experimental approach. *Slc2a2*^*loxP/loxP*^ mice were injected into the 3V with a control viral vector AAV-*Gfap*-GFP or a viral vector that expresses the CRE recombinase under the control of GFAP promoter (AAV-*Gfap*-Cre-GFP). (**B**) Structure of the *Glut2* floxed construction and genomic PCR of mice injected with AAV-*Gfap*-GFP (lane 1) or AAV-*Gfap*-Cre-GFP (lane 2). (**C**) GFP fluorescence (green) in coronal sections (20 µm) of mice transduced for two weeks. DAPI was used as a nuclear marker (magenta). (**D**) GFP fluorescence in GFAP-expressing tanycytes was analyzed through vimentin (purple) and GFAP immunoreactivity (yellow). DAPI was used as a nuclear marker (blue). (**E**) Percentage of GFAP-expressing tanycytes positives for the GFP fluorescence. (**F**) GFP fluorescence in GFAP-expressing tanycytes analyzed through the GFAP immunoreactivity (red). DAPI was used as a nuclear marker (blue). (**G**–**L**) Percentage of GFAP-expressing tanycytes transduced in the hypothalamic anteroposterior and dorsoventral axis (n = 3 mice). *P1* primer 1, *P2* primer 2, *P3* primer 3, *3V* third ventricle, *ME* median eminence, *DMN* dorsomedial nucleus, *VMN* ventromedial nucleus, *ARC* arcuate nucleus, *PVN* paraventricular nucleus, *DTM* dorsal tuberomammillary nucleus.

To delete the *Glut2* gene in GFAP-positive tanycytes from zones 1 to 6, *Slc2a2*^*loxP/loxp*^, mice were stereotactically injected in the 3V with adeno-associated virus (AAV)-*Gfap*-Cre-GFP at bregma AP − 1.82 mm, where the GFAP-expressing tanycytes are projecting to DMN, VMN, and ARC in similar proportions (Fig. 3A). Injection of the AAV-*Gfap*-CRE-GFP induced the expected recombination of the *Glut2* allele, as shown by a 326 bp PCR product (Fig. 3B and Supplementary Fig. 3). The presence of the non-recombined floxed allele corresponding to the 281 bp product was due to the existence of non-transduced cells in this region. To confirm that recombination occurred exclusively in tanycytes, the GFP signal and the percentage of GFAP/GFP-positive tanycytes in the hypothalamus of mice transduced for 2-weeks were evaluated through confocal microscopy. As expected, GFP was detected in the apical region and processes of tanycytes (Fig. 3C, arrowheads). Moreover, GFP fluorescence (green) was observed in GFAP-positive tanycytes (yellow, arrow) that contain endfeet with button morphology, which closely contact other cells present in the hypothalamic parenchyma (Fig. 3D, arrowhead). To determine the percentage of transduction, the number of GFAP-expressing tanycytes positive for GFP fluorescence was quantified. As observed in Fig. 3E, the percentage of GFAP-expressing tanycyte positives for GFP fluorescence was close to 28.5%. In all slices analyzed we detected only one GFAP positive astrocyte transduced.

Since the processes of GFAP-expressing tanycytes contact different nuclei in the anteroposterior and dorsoventral axes, we next evaluated the proportion of transduced cells on both axes using GFP fluorescence. The transduced cells correspond to GFAP-positive tanycytes and their processes extending toward the ARC, ARC/VMN, and DMN, respectively (Fig. 3F, arrowheads). While GFP fluorescence was poorly present in the GFAP-expressing tanycytes in zone 1 (Fig. 3G), it was detected from zones 2 to 6 (Fig. 3H–L). Of the transduced GFAP-positive tanycytes located in zone 2, 25% and 75% contact the ARC and VMN, respectively (Fig. 3H). At the middle region of the hypothalamus, transduced GFAP-positive tanycytes located in zone 3 trace their processes to the ARC, VMN, and DMN in similar proportions (Fig. 3I), while 27.9% and 71.9% GFAP-positive tanycytes located in zone 4 contact the DMN and ARC, respectively (Fig. 3J). At the more posterior region of the hypothalamus, all GFAP-positive transduced tanycytes contact the ARC (Fig. 3K,L). High magnification images (Fig. 3F, inset) GFP-fluorescence was not detected in GFAP-positive cells located in other circumventricular organs, such as the area postrema (Supplementary Fig. 4A–E).

### GFAP-expressing tanycytes regulate feeding behavior through *Glut2*

**Figure 4:**
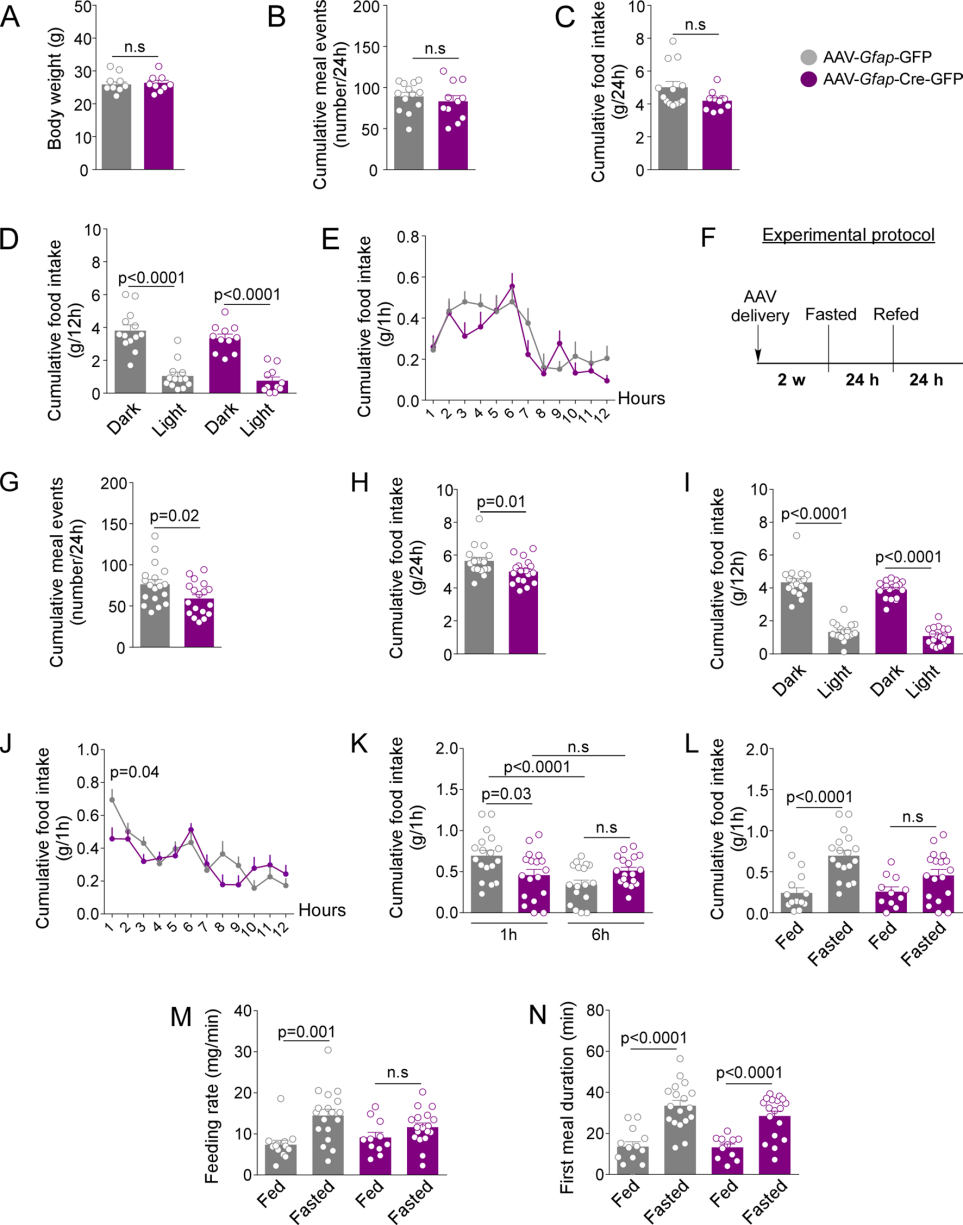
*Glut2* expression in GFAP-expressing tanycytes is required for stimulating feeding in response to fasting. (**A**) Body weight (g), (**B**) cumulative meal events (events/24 h), (**C**) cumulative food intake (g/24 h), (**D**) cumulative food intake (g/12 h) in the dark and light phase of feeding and (**E**) cumulative food intake (g/1 h) during the 12 h of the dark phase of feeding in *Slc2a2*^*loxP/loxP*^ mice transduced with AAV-*Gfap*-GFP or AAV-*Gfap*-Cre-GFP. The parameters were analyzed in basal conditions. (**F**) Experimental approach. (**G**) cumulative meal events (events/24 h), (**H**) food intake (g/1 h), (**I**) cumulative food intake (g/12 h) during the dark and light cycle, (**J**) cumulative food intake (g/1 h) after 24 h of fasting, (**K**) cumulative food intake at 1 h and 6 h of refeeding, (**L**) cumulative food intake (g/1 h), (**M**) feeding rate (mg/min), and (**J**) first meal duration (min) by mice with ad libitum access to food as well as those undergoing a fasting-refeeding period. All the experiments were performed in *Slc2a2*^*loxP/loxP*^ mice transduced for 4-weeks with the viral vector AAV-*Gfap*-GFP or AAV_5_-*Gfap*-Cre-GFP. Error bars represent SEM. Comparisons between two groups were performed using a student’s *t*-test. Multiple comparisons were performed using a two-way ANOVA (Bonferonni’s post-hoc test). *n.s* not significant.

We next evaluated if GLUT2 expressed in GFAP-positive tanycytes is necessary to regulate feeding behavior and energy balance. Feeding behavior in a 24 h feeding cycle (07:00 p.m.–07:00 p.m.) was first evaluated. Body weight, cumulative meal events, and cumulative food intake in 24 h were similar between the control and treatment groups (Fig. 4A–C). Moreover, no significant differences were observed during the 12 h of dark and 12 h of light phase of feeding (Fig. 4D). To more exhaustively analyze the feeding pattern in the 12 h dark cycle, the amount of food consumed every 1 h was evaluated. No significant differences were observed in the meal pattern between experimental groups (Fig. 4E). To further explore whether *Glut2* inactivation in GFAP-positive tanycytes can impact satiety and satiation in basal conditions, we evaluated the mean meal events duration (min), mean events interval duration (min), meal size (g/event), latency of the first meal (min), first meal duration (min), and eating rate (mg/min) parameters. No statistically significant differences were observed in any of the parameters mentioned (Supplementary Fig. 5A–F). Taken together, the results suggest that GLUT2, expressed in GFAP-positive tanycytes, is not necessary to regulate the feeding behavior under basal conditions.

Subsequently, we evaluated the feeding behavior of *Glut2*-inactivated mice in response to fasting. The feeding behavior was evaluated through a fasting-refeeding protocol (24 h/24 h) (Fig. 4F). Interestingly, *Glut2* inactivation in GFAP-positive tanycytes generated a significant decrease in cumulative meal events (events/24 h) and cumulative food intake (g/24 h) (Fig. 4G,H). To determine if *Glut2* inactivation affects food consumption during the dark and light phases, we evaluated the food consumption during the 12 h of the night cycle and the 12 h of the day cycle. As expected, the amount of food consumed is significantly higher in the dark phase than the light phase in both groups (Fig. 4I), which suggests that while the *Glut2* inactivation affects the total food consumption, it does not alter the circadian rhythm of feeding.

Our previous results show that GLUT2 inhibition, in all type of tanycytes, increases total food intake^4^. We next performed a detailed evaluation in the dark phase. Data showed that GLUT2 inactivation led to minor food consumption during the first hour of feeding (Fig. 4J), suggesting that GLUT2 expressed in GFAP-positive tanycytes could regulate the beginning of feeding. To test this hypothesis, we compared the total food consumption during hours 1 and 6 in the dark phase. As observed in Fig. 4K, the control group had a normal response to fasting, presenting a peak in food intake at hour 1 of feeding and significantly decreasing the food consumption at hour 6 of the cycle, indicating that after 6 h feeding the control group is satiated. However, the treated group consumed an amount of food similar during the hour s1 and 6 of the dark phase of feeding, indicating that *Glut2* gene inactivation affects the feeding initiation following a fasting period (Fig. 4K). To confirm that *Glut2-*inhibited mice did not show a response to negative energy balance, we compared the cumulative food intake, the eating rate (mg/min), and the duration of the first feeding (min) during the first hour of the dark phase (g/1 h) after 24 h fasting or in mice with ad libitum access to food. As seen in Fig. 4L, the control group with ad libitum feeding had a significant increase in the cumulative food intake in response to 24 h of fasting. Nevertheless, this response to fasting is lost in *Glut2*-inactivated mice (Fig. 4L). Moreover, *Glut2* gene inactivation decreased the eating rate in response to fasting (Fig. 4M). However, we did not observe a significant difference in the first meal duration of the treated group between both conditions (Fig. 4N).

To further explore if these results were related to altered satiety, we analyzed the following parameters: mean meal events duration (min), mean events interval duration (min), meal size (g/events), the latency of the first meal (min), first meal duration (min), and eating rate (mg/min). No significant difference was observed between the control and treated groups in any of the parameters analyzed (Supplementary Fig. 5G–L), suggesting that the inactivation of *Glut2* does not affect satiety. Altogether, the data strongly suggest that a GLUT2-dependent mechanism in GFAP-positive tanycytes is necessary to stimulate feeding and regulate the feeding initiation following a fasting period.

### GLUT2 expression in GFAP-tanycytes is required to control the ghrelin secretion

**Figure 5:**
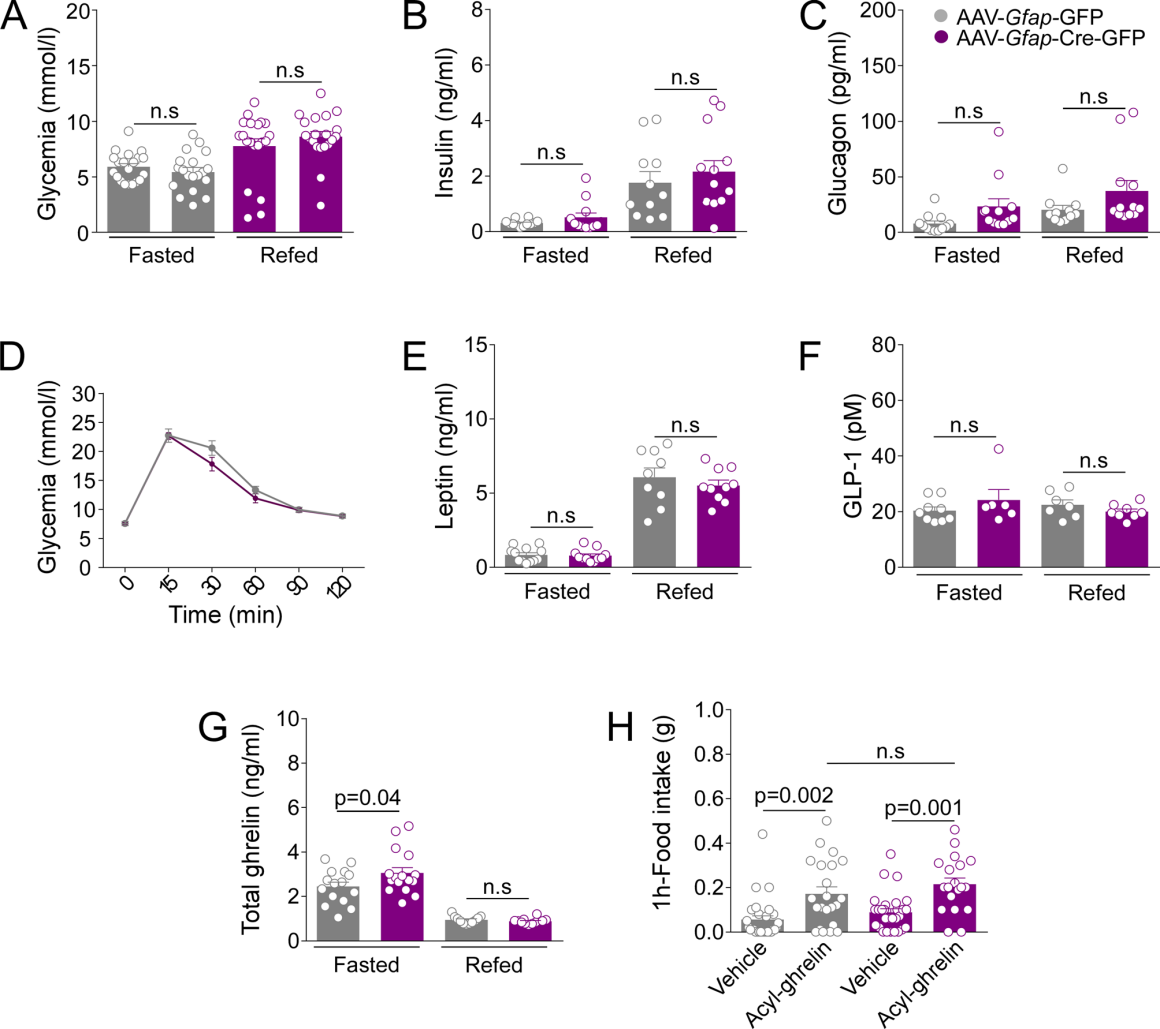
*Glut2* inactivation enhances the ghrelin secretion in fasting. (**A**) Blood glucose concentration (mmol/L), (**B**) plasma insulin concentration (ng/mL) and (**C**) plasma glucagon concentration (pg/mL). (**D**) Mice show a normal i.p GTT (2 g/kg). (**E**) Plasma leptin (ng/mL), (**F**) plasma GLP-1 (pM) and (**G**) plasma total ghrelin (ng/mL) concentrations. All the measurements were performed on mice transduced for 4-weeks with the viral vector AAV-*Gfap*-GFP (grey bars) or AAV-*Gfap*-Cre-GFP (purpure bars). Plasma samples were obtained after 24 h of fasting and 6 h of refeeding. (**H**) Ghrelin response. Total food intake (g) was measured 1 h post i.p treatment of mouse acyl-ghrelin (10 µg/kg) or vehicle (NaCl 0.9% w/v). Error bars represent SEM. Multiple comparisons were performed using a two-way ANOVA (Bonferonni’s post-hoc test). *n.s* not significant.

In vivo studies suggest that GLUT2 expression in the CNS is necessary to control the secretion of hormones controlling glucose homeostasis, such as insulin and glucagon^5,9^. With this in mind, we evaluated whether GLUT2 must be expressed in GFAP-positive tanycytes to maintain normal blood values of glucose and peripheral hormones in fasted and refed mice. Glycemia, insulin, and glucagon at 24 h fasting and 6 h refeeding were similar in control and *Glut2*-inactivated mice (Fig. 5A–C). Concurrently, glucose tolerance tests (GTT) did not show any significant differences between the control and treated groups, indicating that the *Glut2* deletion in GFAP-positive tanycytes did not alter glucose homeostasis (Fig. 5D).

Studies conducted in rip*glut1*;*glut2*^−/−^ mice showed that GLUT2 expression is required to control the peripheral ghrelin secretion during the fasting-to-refeeding transition^3^. Therefore, the effect of *Glut2-*gene inactivation on ghrelin, leptin and GLP-1 secretion was evaluated. No significant difference was observed in the plasma levels of leptin and GLP-1 between the control and treated groups (Fig. 5E,F). Nevertheless, we detected abnormal plasma levels of the orexigenic hormone ghrelin in the treated group (Fig. 5G). As expected, after 24 h fasting, total ghrelin was high and decreased significantly after 6 h refeeding in the control and treated groups. However, *Glut2*-inactivated mice showed significantly higher total ghrelin values than control at 24 h of fasting (Fig. 5G). When acylated ghrelin was i.c.v. injected, a similar increase in food intake was detected (Fig. 5H). These results indicate that the secretion of total-ghrelin in response to fasting is partly regulated in the hypothalamus by a GLUT2-dependent mechanism involving GFAP-positive tanycytes.

### Genetic inactivation of *Glut2* increases the c-Fos expression in the VMN

**Figure 6:**
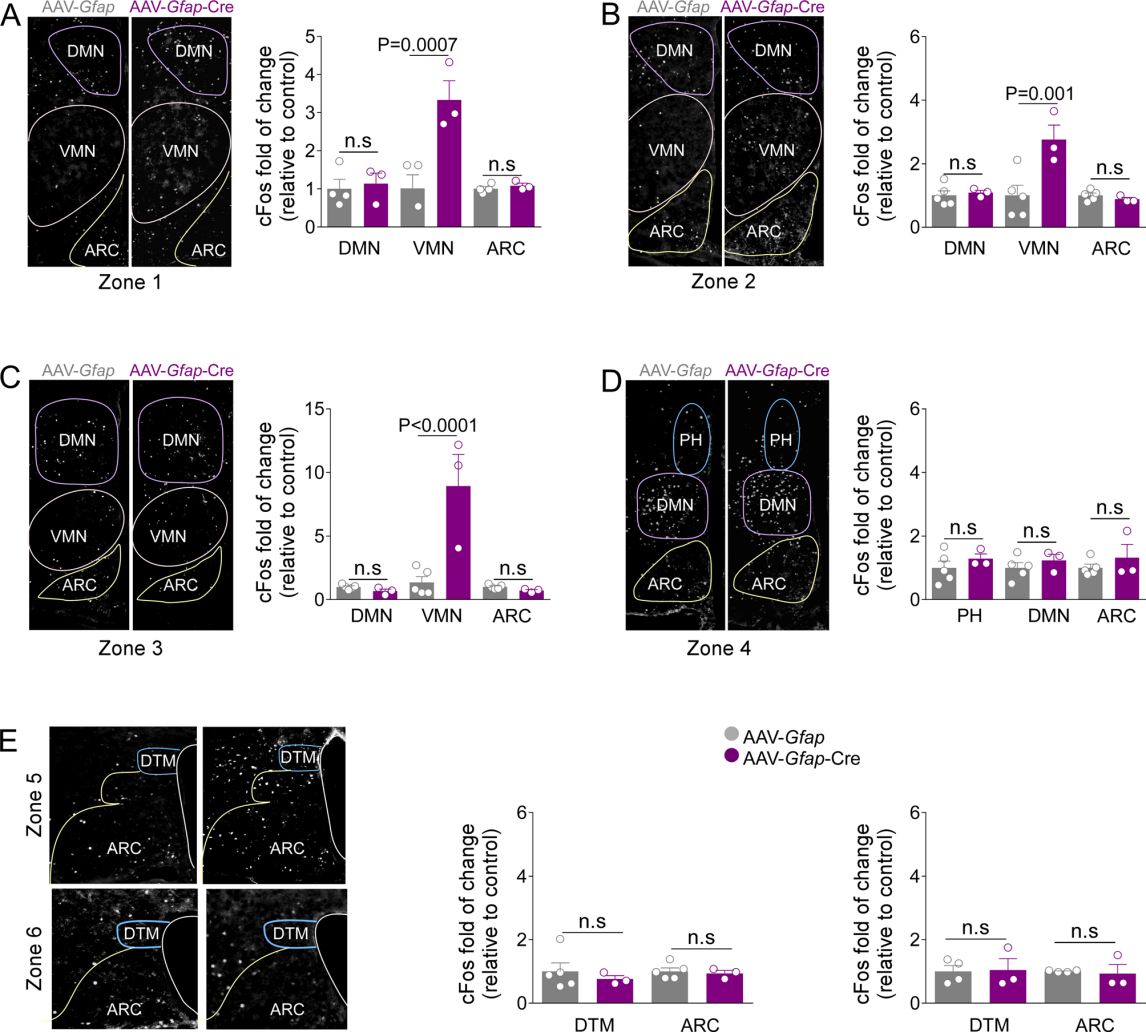
*Glut2* inactivation increases c-Fos expression in the VMN response to fasting. (**A**–**E**) Representative images and quantification of c-Fos-immunoreactive cells (white) in the hypothalamus of *Slc2a2*^*loxP/loxP*^ mice transduced for 4-weeks with the viral vector AAV-*Gfap*-GFP (grey bars) or AAV-*Gfap*-Cre-GFP (purpure bars). Antero-posterior c-Fos quantification was performed from the bregma AP-1.54 mm (**A**) to bregma AP − 2.54 mm (**E**) in 24 h fasted mice. Error bars represent SEM. Multiple comparisons were performed using a two-way ANOVA (Bonferonni’s post-hoc test). *DMN* Dorsomedial nucleus, *VMN* ventromedial nucleus, *ARC* arcuate nucleus, *DTM* dorsal tuberomammillary nucleus, *PVN* paraventricular nucleus, *3V* third ventricle. *n.s* not significant.

We previously showed that *Glut2* inactivation affects feeding exclusively in response to fasting. Therefore, to determine whether GFAP-expressing tanycytes regulate the activity of neighboring neurons through GLUT2, we evaluated c-Fos activation in response to a 24 h fasting. As seen in Fig. 6A,B, *Glut2* inactivation generated a significant increase in c-Fos expression in the VMN in zone 1 and zone 2 of the hypothalamus, without impacting DMN and ARC activation. Moreover, in zone 3, *Glut2* inactivation generated a large decrease in c-Fos-positive cells number in the DMN and a significant increase in the VMN without impacting c-Fos expression in the ARC (Fig. 6C). Conversely, no significant changes in the expression of c-Fos were detected in zones 4–6 compared to the control group (Fig. 6D,E). Together, these findings suggest that GFAP-positive tanycytes regulate neuronal activation in response to fasting through a GLUT2-dependent mechanism mainly in the VMN.

### *Glut2* inactivation in GFAP-expressing tanycytes disturbs *Pomc* gene expression

**Figure 7:**
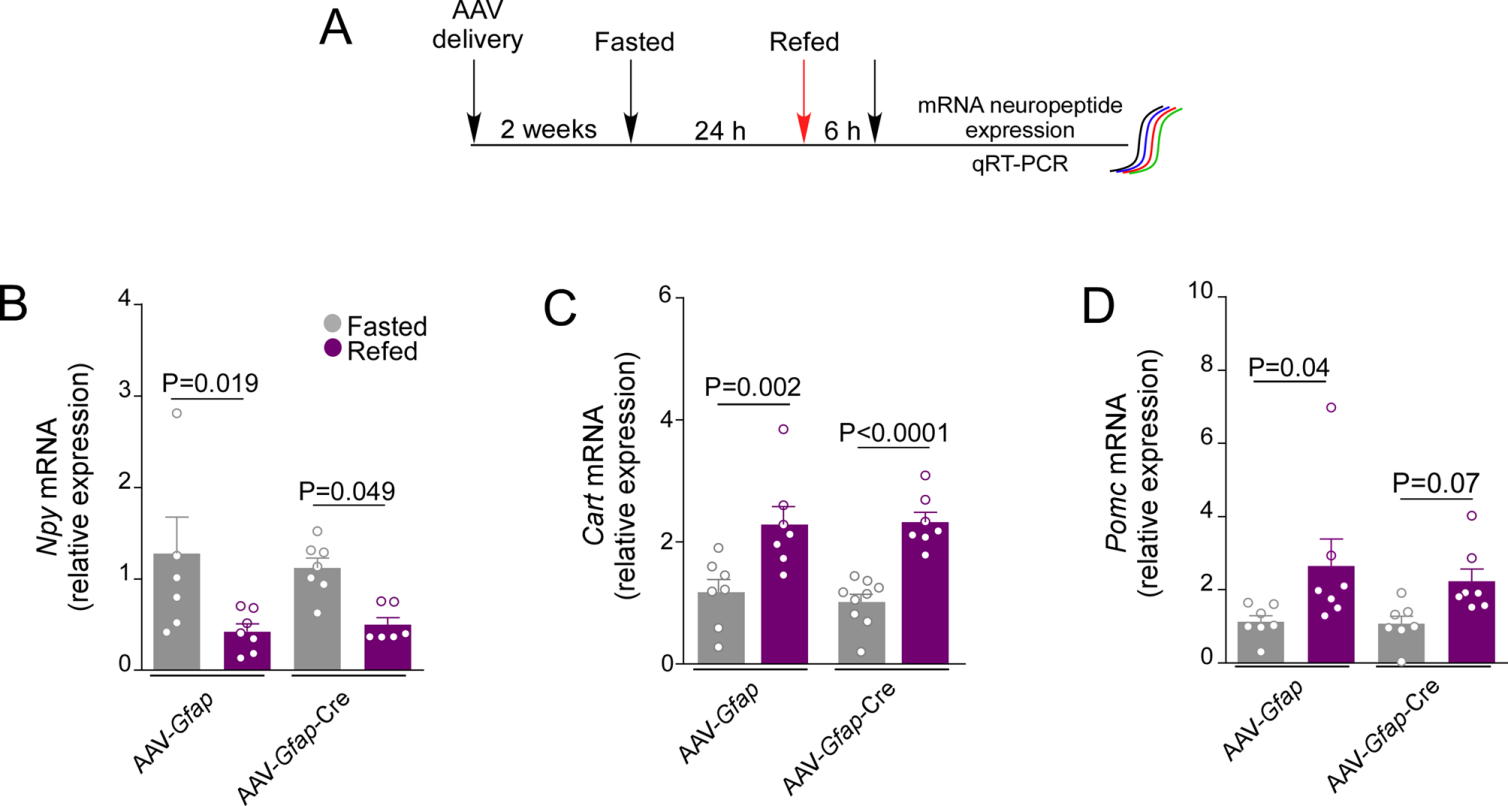
Loss of regulated *Pomc* expression in response to *Glut2* inactivation during the fasting-to-refeeding transition. (**A**) Experimental approach. *Slc2a2*^*loxP/loxP*^ mice transduced for 6-weeks with the viral vector AAV-*Gfap*-GFP or AAV-*Gfap*-Cre-GFP. Total RNA was obtained after 24 h of fasted (grey bars) and 6 h of refed (purpure bars). Analysis of *Npy* (**B**), *Cart* (**C**), and *Pomc* (**D**) mRNA expression using qRT‐PCR. Error bars represent SEM. Multiple comparisons were performed using a two-way ANOVA. *n.s* not significant.

To determine if c-Fos activation affects orexigenic or anorexigenic neurons, the genetic expression of neuropeptides, *Npy*, *Cart,* and *Pomc,* was evaluated in response to a 24-h fasting and 6-h refeeding period (Fig. 7A). In control mice, the orexigenic neuropeptide, *Npy,* was high after a 24 h fasting, decreasing significantly after the 6 h of refeeding (Fig. 6B). Interestingly, in *Glut2-*inactivated mice, *Npy* mRNA (Fig. 7B) was not altered. Regarding anorexigenic neuropeptides, the levels of *Cart* mRNA in response to 6 h of feeding were increased as expected in both groups (Fig. 7C), whereas the loss in the POMC normal response to refeeding was observed in *Glut2*-inactivated mice (Fig. 7D). Altogether, the results indicate that GFAP-expressing tanycytes modulate the gene expression of POMC neurons during the fasted-to-refeeding transition.

## Discussion

The present work provides new evidence on the role of tanycytes in the regulation of food intake. At the same time, through detailed neuroanatomical analysis, we show that GFAP-expressing tanycytes are a heterogeneous population. The number, location, and contacts with different hypothalamic nuclei of GFAP-expressing tanycytes vary severely in the dorsoventral and anteroposterior axis.

Tanycytes have been historically classified based on three criteria: the location in the dorsoventral axis, the extension of the processes towards the hypothalamic parenchyma, and the expression of immunohistochemical markers^24^. Therefore, tanycytes are exclusively categorized according to their dorsoventral axis, and their location pattern on the anteroposterior axis remains largely unknown. Although this classification has been widely used for many years, several works suggest that it should be modernized and redefined^25–27^.

All tanycyte populations express common genetic markers, such as *Vim*, *Sox2,* and *Slc2a1*^17,22,23^. *Gfap* expression has also been reported in α-tanycytes^22,23^, as well as in dorsal β1-tanycytes^28^. The present study is consistent with this, detailing the localization of GFAP-expressing tanycytes along the anteroposterior axis for the first time. Through 3D analysis, we showed that GFAP-positive and GFAP-negative tanycytes are asymmetrically distributed along the anteroposterior axis into the 3V and face the DMN, VMN, and ARC nuclei. GFAP-positive tanycytes are mainly located in the anterior hypothalamic area, establishing a more accentuated functional morphological relationship with DMN and VMN. In this context, our study supports the hypothesis that tanycytes establish heterogeneous contacts, with various neuronal nuclei so they can fulfill different functions in hypothalamic control of energy balance. In this context, our study supports the hypothesis that tanycytes are a fairly heterogeneous population that, depending on their location, can fulfill different functions in hypothalamic control of energy balance.

Injection of AAV-*Gfap-*Cre-GFP in mouse *Slc2a2*^*loxP/loxP*^ expectedly inactivated GLUT2 expression by tanycytes. Nevertheless, not all GFAP-expressing tanycytes located in the different hypothalamic zones were infected in the same proportion. We speculated that low efficiency of the AAV-virus could be due to technical limitations. The AAV-*Gfap*-Cre-GFP was injected in the medial region of the hypothalamus located in the bregma AP: − 1.82 mm, an area in which GFAP-expressing tanycytes contact the DMN, VMN, and ARC in the same proportion. Given that the CSF circulates in an anteroposterior direction, and we injected 0.5 µL, the volume used may not be enough to transduce the GFAP-expressing tanycytes located in the bregma AP: − 1.34 mm. Although the number of transduced tanycytes was low, it was sufficient to induce changes in murine feeding behavior in response to fasting, confirming the importance of GLUT2 and the role of the hypothalamic glia in this circuit. Even though it was possible to detect transduction in subependymal astrocytes because the AAV used is under the control of a GFAP promoter, our results agree with previous reports that astrocytes express GLUT1^29^ and not GLUT2^10^.

Here, we showed that *Glut2*-inactivation in mice generated a decrease in food intake and a delay in the feeding initiation, only in the context of extended fasting conditions. Interestingly, the delay in feeding initiation after a fasting period was also observed by Bady^3^ and Rohrbach et al.^27^. Using a GLUT2 knockout model, Bady et al.^3^ showed that the GLUT2 deletion delays meal initiation after a 24-h fasting period. Similarly, Rohrbach et al.^27^ showed that the specific tanycyte ablation in mice, induced by the genetic deletion of glucokinase, induces a delay in meal initiation after fasting. It is important to highlight that, although our work shows that the alterations occur under negative energy balance conditions, the signal sent by tanycytes to neurons in response to hypoglycemia has not been elucidated. Nevertheless, we showed that GFAP-positive tanycytes primarily contact the VMN, a crucial hypothalamic region known as the satiety center^30,31^. Furthermore, we determined that *Glut2* inactivation in GFAP-positive tanycytes increases the number of c-Fos-positive cells in the VMN in response to fasting. Interestingly, selective activation of the SF1 neuron population in the VMN, using DREADD (designer receptors exclusively activated by designer drugs) technology, can suppress food intake^32^. Accumulating evidence indicates that SF1 neuronal activation in fasted SF1-hM3Dq mice suppressed feeding during the first hours of the refeeding cycle^33^, showing a delay in the meal initiation similar to observed in the *Glut2*-inactivated mice. VMN SF1 neurons form the majority of VMN neurons and are glucose-responsive^34^. Based on these findings, it is tempting to speculate that the genetic inactivation on *Glut2* in GFAP-expressing tanycytes generates a hyperactivation of VMN SF1 neurons (in hypoglycemia condition), leading to suppression of feeding after fasting. However, we need to conduct more studies to determine the complexity of this relationship.

In addition to its role in controlling satiety, the VMN plays an important role in regulating sympathetic nerve activity (SNA) and counterregulatory responses to hypoglycemia^35,36^. Several studies have shown a close relationship between the VMN and SNA output^37,38^. In this context, it was demonstrated that the SNA stimulates ghrelin secretion in response to fasting^39–41^. Therefore, it is possible to speculate that *Glut2*-inactivated mice generated hyperghrelinemia in response to fasting via the VMN-> SNA axis. Our results agree with previous studies in GLUT2 knockout mice, showing that this glucose transporter is necessary to regulate plasma ghrelin secretion during the fasting-to-refeeding transition^3^. It is important to mention that our results show that hyperghrelinemia, observed in *Glut2-*inactivated mice, did not stimulate food intake in response to fasting. Consistent with this finding, ablation of ghrelin cells in adult mice does not decrease appetite or body weight in the short or long term^42^, suggesting that it is not essential for feeding behavior control in mice but is required for other functions, such as lipid storage^43,44^ or glucose homeostasis regulation^45^.

In contrast to what we have previously reported in rats^4^, inactivation of *Glut2* in mice only generated changes in *Pomc* gene expression during the refeeding cycle. This differential regulation is likely due to the type of contact between GFAP-expressing tanycytes processes and POMC neurons. In the ARC, NPY, and POMC neurons have a segregated distribution^46^, NPY neurons have a proximal location to the 3V, while POMC neurons have a distal site^47,48^. GFAP-expressing tanycyte processes mainly contact the most distal region of the ARC, while β1-tanycytes mostly approach the proximal ventricular region. Therefore, GFAP-expressing tanycytes may control the activity of POMC neurons. In POMC neurons it has been demonstrated that lactate supply by tanycytes sustains the activity of POMC neurons^49,50^. However, we cannot rule out that other peripheral signals or another neuronal input (e.g., axons coming from VMN^51^) or other nuclei may regulate this event. Further studies are required for understand this apparent controversial response. Additionally, it is important to mention that our study also has limitations. For all our approaches, only male mice were used, while female mice were excluded from any experimental phase. Therefore, more information is required to determine if what was observed in our work is also replicated in females.

In conclusion, GFAP-expressing tanycytes are a subpopulation of cells with a different role than GFAP-negative tanycytes, which are preferentially located in the anterior portion and establish contacts with DMN and VMN. GFAP-positive tanycytes that express GLUT2 play a role in normal food intake main during fasting.

## Material and methods

### Mice

All experiments were reviewed and approved by the Animal Ethics Committee of the Chilean National Commission for Scientific and Technological Research (CONICYT, the protocol for projects #1221508) and the veterinary office of the Canton de Vaud (Switzerland). Mice were treated in compliance with the U.S. National Institutes of Health guidelines for animal care and use. All methods of this study were carried out in accordance with ARRIVE guidelines (https://arriveguidelines.org, accessed on 1 July 2021). C57BL/6J and *Slc2a2*^*loxP/loxP*^ male mice strains were used. *Slc2a2*^*loxP/loxP*^ mice were generated as previously described^52^. Experiments were performed with a total of 50 male mice from 8- to 12-week-old. For all the experiments, mice were randomly assigned to experimental groups. No blinding was done in the assignment of groups and data analysis. Mice were housed 4–5 per cage at 23 °C using a 12 h light/dark cycle (07:00 a.m. on, 07:00 p.m. off). All animals had ad libitum fed with a standard rodent chow diet (Diet 3436, Provimi Kliba AG, Kaiseraugst, Switzerland), except under experimental conditions. The study was not pre-registered; all relevant information is provided in the manuscript and custom-made materials will be provided upon request.

### AAV

The viral constructs were obtained from the Vector Core of the Gene Therapy Center at the University of North Carolina (UNC, North Carolina, USA). To inactivate the *Glut2* expression in vivo, the associated-adenovirus (AAV) AAV5-Gfap-GFP-Cre (4.9 × 10^12^ particles/ml) was used. As a control, the AAV5-Gfap-GFP (4.9 × 10^12^ particles/ml) (UNC Vector Core, North Carolina, USA) was used. Plasmid pAAV-Gfap-GFP-Cre is 6550 bp in size, of which the GFAP promoter is 690 bp in size. The EGFP and Cre sequence are linked by the autocleving side. Additionally, the commercial plasmid contains the Woodchuck Hepatitis Virus (WHP) Posttranscriptional Regulatory Element (WPRE), which is a DNA sequence that enhancing gene expression.

### AAV delivery

Randomly chosen *Slc2a2*^*loxP/loxP*^ mice were anesthetized with an intraperitoneal (i.p.) injection of ketamine (90 mg/kg) and xylazine (10 mg/kg). After checking the post-anesthesia absence of reflex, mice were positioned in the stereotactic frame (Stoelting, Wood Dale, USA). Mice were maintained at 37 °C throughout the surgery. *Glut2* genetic inactivation, in GFAP-tanycytes, was induced through the infusion of 0.5 µL (0.1 μL min^−1^) of AAV_5_-*Gfap*-Cre-GFP (4.9 × 10^12^ particles/mL) (UNC Vector Core, North Carolina, USA) into AP − 1.8 mm, ML: 0.0 mm, DV: − 5.5 mm. Because the model generated is permanent and not transient, once the silencing is produced (2 weeks), the experiments were conducted after 2- to 4-week of transduction. The sites of injection were verified by GFP fluorescence and the percentage of GFAP/GFP tanycytes on 20 µm brain sections.

### Immunohistochemistry

Mice were transcardially cannulated and perfused with 4% paraformaldehyde (PFA). Subsequently, the samples were soaked in a 30% sucrose solution for 72 h following the protocol described^53^. Brains were cut into 20-µm sections with a cryostat (Microm HM520), and sections were incubated with the primary antibodies for 16 h at 4 °C. Rabbit anti-GLUT2 (1:200; Alomone, AGT-022, Jerusalem BioPark, Jerusalem, Israel), chicken anti-vimentin (1:400; Millipore, AB5733, Billerica, MA, USA), and mouse anti-GFAP (1:500; Millipore, MAB360) were used as primary antibodies after dilution in PBS and 1% bovine serum albumin. After washing, sections were incubated for 2 h at room temperature with Alexa-Fluor 488-labeled secondary antibody (1:200, A11008, Invitrogen Thermo Fisher, Massachusetts, USA), Alexa-Fluor 568-labeled secondary antibody (1:200, A-11031, Invitrogen Thermo Fisher), and Alexa-Fluor 647-labeled secondary antibody (1:200 A-21449, Invitrogen Thermo Fisher). DAPI (1:1000; Invitrogen Thermo Fisher) was used as a DNA stain. Sections were analyzed using an Axio Imager.Z1 ApoTome microscope (Zeiss, Germany) and confocal‐spectral laser microscopy (LSM 780 NLO, Zeiss). 3D-GLUT2 reconstruction was processed using the IMARIS FilamentTracer (Oxford Instruments, Concord, MA).

### Genomic PCR

At 2-weeks post-transduction of *Glut2* genetic inactivation, mice were anesthetized with isoflurane and sacrificed by cervical dislocation. Genomic DNA (gDNA) was extracted from the hypothalamus following the manufacturer’s instructions (Quick-DNA kit, Zymo research, LucernaChem AG, Luzern, Switzerland) and stored at − 80 °C for later use. gDNA amplification by PCR was performed using hot start Taq DNA polymerase (Intact Genomics, St. Louis, Switzerland) and the following primers: P1: 5′-CCA ATC CCT TGG TTA TGG TTG C-3′, P2: 5′-CGT AAG GCC CAA GGA AGT CCT GC-3′ and P3: 5′-CTG CTA AAG CGC ATG CTC CAG AC-3′. PCR amplification was made using the following PCR program: 95 °C for 3 min; 95 °C for 15 s; 62 °C for 15 s; 72 °C for 15 s; 72 °C for 1 min; 35 cycles.

### Neuropeptide mRNA expression analysis

*Slc2a2*^*loxP/loxP*^ transduced mice were fasted for 24 h or fasted and refed for 6 h, anesthetized using isoflurane, and sacrificed by decapitation. The brains were delicately removed, and the hypothalamus dissection was performed in a cold medium containing artificial cerebrospinal fluid (119 mM NaCl, 26.2 mM NaHCO_3,_ 2.5 mM KCl, 1 mM NaH_2_PO_4,_ 1.3 mM MgCl_2_). Quickly, the samples were frozen in liquid nitrogen and stored at − 80 °C. Total RNA was isolated using the TriFast method, and cDNA was synthesized from 1 µg total RNA with random hexamers (Applied Biosystems, Zug, Switzerland) and M-MLV reverse transcriptase (Promega, USA) following the manufacturer’s instructions. qRT-PCR reactions were prepared using SYBR Green Master Mix (Applied Biosystems). The following sets of primers (forward: F and reverse: R) were used: F5′-ACG TGG AAG ATG CCG AGA TT-3′ and R5′-CAA ACC AAG GTG GTG TCC GT-3′ for *Pomc*, F5′-CAG AGT TCC TCA GGT CTA AGT C-3′ and R5′-TTG AAG AAG CGG CAG TAG CAC-3′ for *AgRP*, F5′-ATG GGG CTG TGT GGA CTG AC-3′ and R5′-AAG TTT CAT TTC CCA TCA CCA C-3′ for *Npy* and F5′-TAC GGC CAA GTC CCC ATG TG-3′ and R5′-GGG GAA CGC AAA CTT TAT TGT TG-3′ for *Cart*, F5′-CGG GAC TTT ATT GGC TGG GT-3′ and R5′-CCT CCC TCA TGT TCC ACC AC-3′ for *GusB*. mRNA level expression was measured in a 7500 Fast Light Cycler System (Applied Biosystems, Zug, Switzerland) using the following program: 95 °C for 10 min; 95 °C for 30 s; 60 °C for 20 s; 72 °C for 20 s; 40 cycles. Neuropeptide mRNA expression was calculated by the comparative CT method using *GusB* as the housekeeping control gene.

### c-Fos analysis

Brains were removed from 24 h fasted mice and processed as described above. Briefly, sections were washed with PBS and incubated 1 h in blocking solution (2% normal goat serum + 0.3% Triton in 1× PBS) at room temperature and then incubated with the primary rabbit anti-cFos antibody (Cell Signaling, 2250S 9F6, Massachusetts, USA) diluted 1:3000 in blocking solution for 24 h at room temperature. After washing, sections were incubated for 1 h at room temperature with a goat anti-rabbit immunoglobulin (IgG) antibody coupled to Alexa-Fluor 488 1:400 (Invitrogen; A11008, Thermo Fisher, MA, USA) prepared in blocking solution. C-Fos quantification was performed using the ImageJ software in 3- to 4-slices per bregma, bilaterally from AP − 1.34 mm to − 2.54 mm coordinates. The number of c-Fos positive cells was averaged for each animal. The data is represented cFos fold of change relative to control.

### Glucose tolerance test (GTT)

Mice were fasted overnight and injected i.p. with a 30% glucose solution (1 μL/g of body weight). The glycemia was measured as described above at 15, 30, 60, 90- and 120-min post-injection. Blood glucose levels were measured in blood drops from the lateral tail vein using an Ascensia Breeze2 glucometer (Bayer Healthcare, Leverkusen, Germany).

### Hormonal assays

Plasma samples were obtained through a submandibular puncture under isoflurane anesthesia at the end of 24 h of fasting and 6 h of refeeding. For acyl ghrelin and total ghrelin essays, plasma was treated with 1 mg/mL pefabloc SC (Sigma-Aldrich, St. Louis, MO, USA) and acidified with 0.05 N HCl. ELISA analyses were performed using the following assays: EZRGRA-90K and EZRGRT-91 K for acyl and total ghrelin (Millipore, Billerica, MA, USA), Ultrasensitive Mouse insulin ELISA (10-1247-01; Mercodia, Winston-Salem, USA), glucagon ELISA (10-1281-01; Mercodia), leptin ELISA (EZML-82K; Millipore) and GLP-1 ELISA (81508; CrystalChem).

### Feeding behavior and ghrelin sensitive test

The mice were habituated to the cages (BioDAQ, Research Diet, US) for 1 week before the experiments were initiated. Daily feeding behavior was measured for 24 h (07:00 p.m.–07:00 p.m.). Fasting/refeeding (24 h/24 h) experiments were performed over a 48-h period (07:00 p.m.–07:00 p.m.). Body weight was measured at the end of the fasting and refeeding condition. Satiety parameters, including mean meal duration (events/min), mean meal side (g/events), eating rate (g/min), latency of the first meal (min), first meal duration (min), and meal interval duration (min), were calculated as previously described^4^. For ghrelin sensitivity test, mice with ad libitum access to food were i.p. injected (08:00 a.m.) with the vehicle (NaCl 0.9% w/v) or mouse acyl-ghrelin (10 µg/kg) (Bachem, Bubendorf BL, Switzerland), and food consumption was measured 2 h later.

### Three-dimensional distribution of GFAP-expressing tanycytes

GFAP and vimentin expression (n = 6 mice, 3 slice per mice) in the ventricular wall was analyzed by immunohistochemistry through the anteroposterior and dorsoventral axis. Three-dimensional analysis was performed from bregma AP − 1.34 to − 2.54 mm^54^. The percentage of GFAP-positive per bregma was obtained as follows: Total GFAP-positive area (µm) × 100/vimentin-positive area (µm). The percentage of GFP-transduced tanycytes that contacts the ARC, VMN, and DMN was calculated from the total number of transduced cells. Three to four slices per bregma were bilaterally quantified using the ImageJ software.

### Statistical analysis

All values were expressed as the mean ± standard error of the mean (SEM). The data was assumed to have a normal distribution. No subjects were excluded from the experiments or the statistical analysis. *t*-tests were used for comparing two groups and ANOVA test (followed by Bonferroni’s post hoc) for multiple comparisons, using GraphPad Prism 5.0 Software (GraphPad Software Inc., San Diego, CA, USA). No randomization was performed to allocate animals in the study. No blinding was performed in the experiments. No sample calculation was performed and the study was exploratory. The number of animals used for each experiment, the statistical test applied and the P-values are described in Supplementary Table 1.

## Supplementary Information

Supplementary Information.

## Data Availability

All original data will be made available upon request to the corresponding authors.
