# Supplementary material for: GLUT2 expression by glial fibrillary acidic protein-positive tanycytes is required for promoting feeding-response to fasting
Source: Sci Rep. 2022 Oct 21;12:17717. doi: 10.1038/s41598-022-22489-2 (PMC9587252; doi:10.1038/s41598-022-22489-2)
Supplement: Supplementary file 1 — Supplementary Information. [file 41598_2022_22489_MOESM1_ESM.pdf]

GLUT2 expression by glial fibrillary acidic protein-positive tanycytes is required for promoting feeding-response to fasting.

Barahona MJ[1,2], Langlet F[3,4], Labouèbe G[3], Croizier S[3], Picard A[3], Thorens B*[3], García-Robles MA*[1,5].

[1]Laboratorio de Biología Celular, Departamento de Biología Celular, Facultad de Ciencias Biológicas, Universidad de Concepción, Concepción, Chile.

[2]Current affiliation: Laboratorio de neurobiología y células madres (NeuroCellT), Departamento de Biología Celular, Facultad de Ciencias Biológicas, Universidad de Concepción, Concepción, Chile.

[3]Center for Integrative Genomics, Faculty of Biology and Medicine, University of Lausanne, Lausanne, Switzerland.

[4]Current affiliation: Department of Biomedical Sciences, Faculty of Biology and Medicine, University of Lausanne, Lausanne, Switzerland.

[5]Instituto de Neurociencias, Centro Interdisciplinario de Neurociencias de Valparaíso, Universidad de Valparaíso, Valparaíso, Chile

Corresponding authors:

María A García-Robles, Departamento de Biología Celular, Facultad de Ciencias Biológicas, Universidad de Concepción, Concepción, Chile. Tel: +56 (41) 2203805, email: mgarcia@udec.cl

Bernard Thorens, Center for Integrative Genomics, Faculty of Biology and Medicine, University of Lausanne, Lausanne, Switzerland. Tel: +41216923980/81, email: bernard.thorens@unil.ch

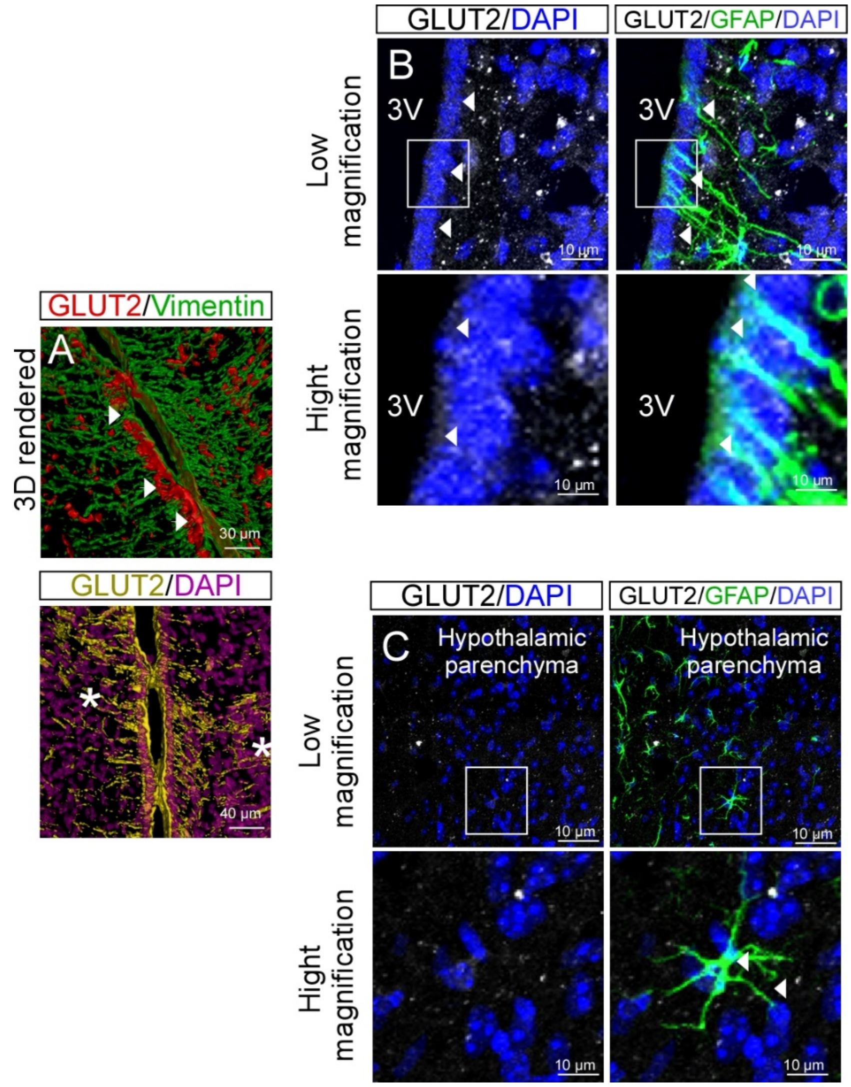

Supplementary Figure 1: GLUT2 expression in the basal hypothalamus.

(A) 3D rendered of tanycytes using anti-GLUT2 (red) and anti-vimentin (green) antibodies. Hight magnification imagen show GLUT2 (yellow) in the apical area. Dapi was used as a nuclear marker (purpure). **(B)** Low and high magnification images of GFAP-positive tanycytes using anti-GLUT2 (white) and anti-GFAP (green) antibodies. **(C)** Low and high magnification images of GFAP-positives astrocytes using anti-GLUT2 (white) and anti-GFAP (green) antibodies. DAPI was used as a nuclear marker (blue). 3V: third ventricle, ME: median eminence, VMN: Ventromedial nucleus, ARC: Arcuate nucleus.

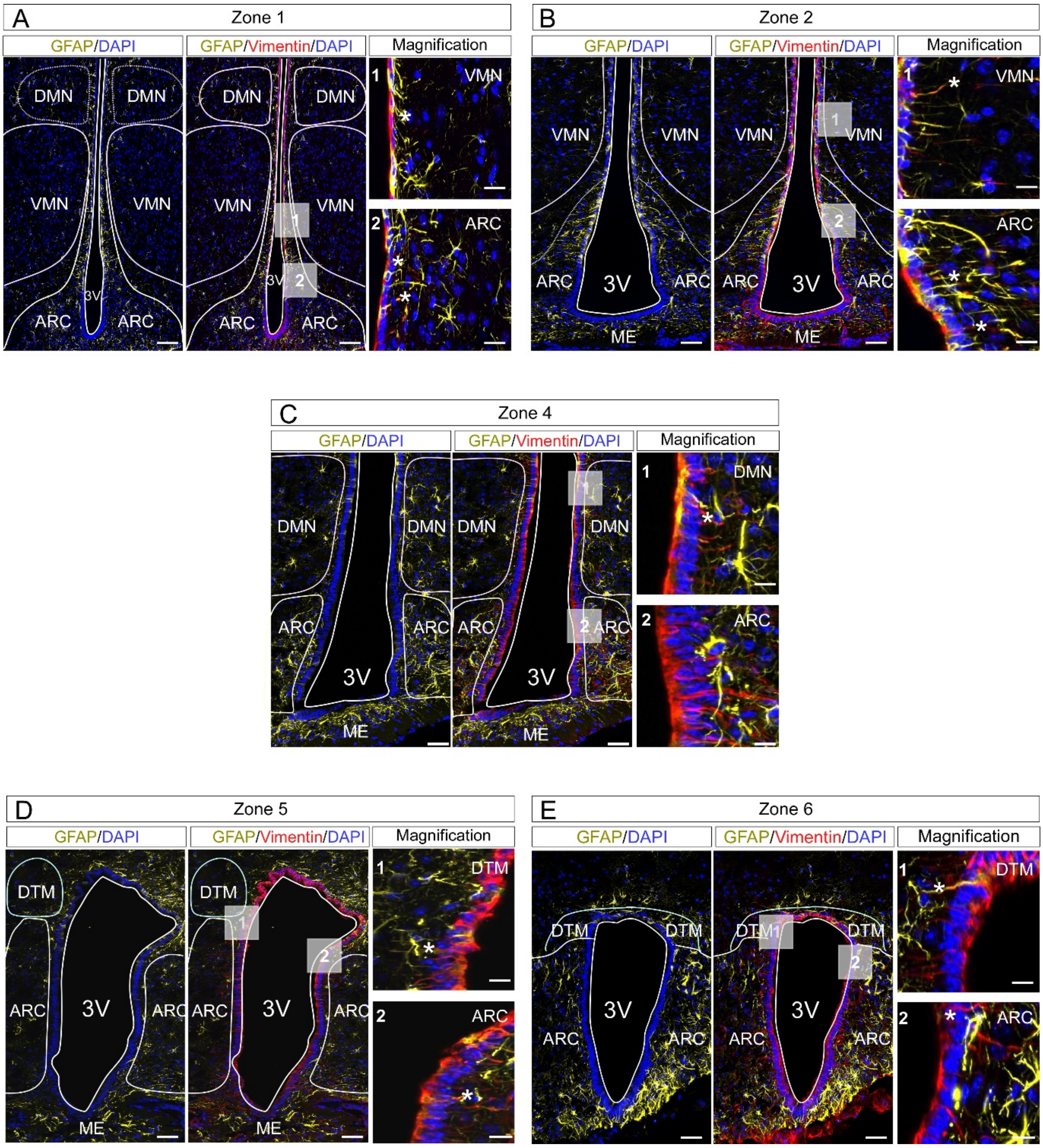

Supplementary Figure 2: Hypothalamic dorsoventral distribution of GFAP-expressing tanycytes.

(A-E) Coronal sections of the hypothalamus using the anti-GFAP (yellow) and anti-vimentin (red) antibodies and the nuclear marker, DAPI (blue). Low and high magnification images were obtained in the hypothalamic region bregma AP -1.34 mm **(A)**, bregma AP -1.58 mm **(B)**, bregma AP -2.06 mm **(C)**, bregma AP -2.30 mm **(D)** and bregma AP -2.54 mm **(E).** 3V:

third ventricle, ME: median eminence, DMN: Dorsomedial nucleus, VMN: Ventromedial nucleus, ARC: Arcuate nucleus, DTM: Dorsal tuberomamilar nucleus. Scale bar: 20 μm.

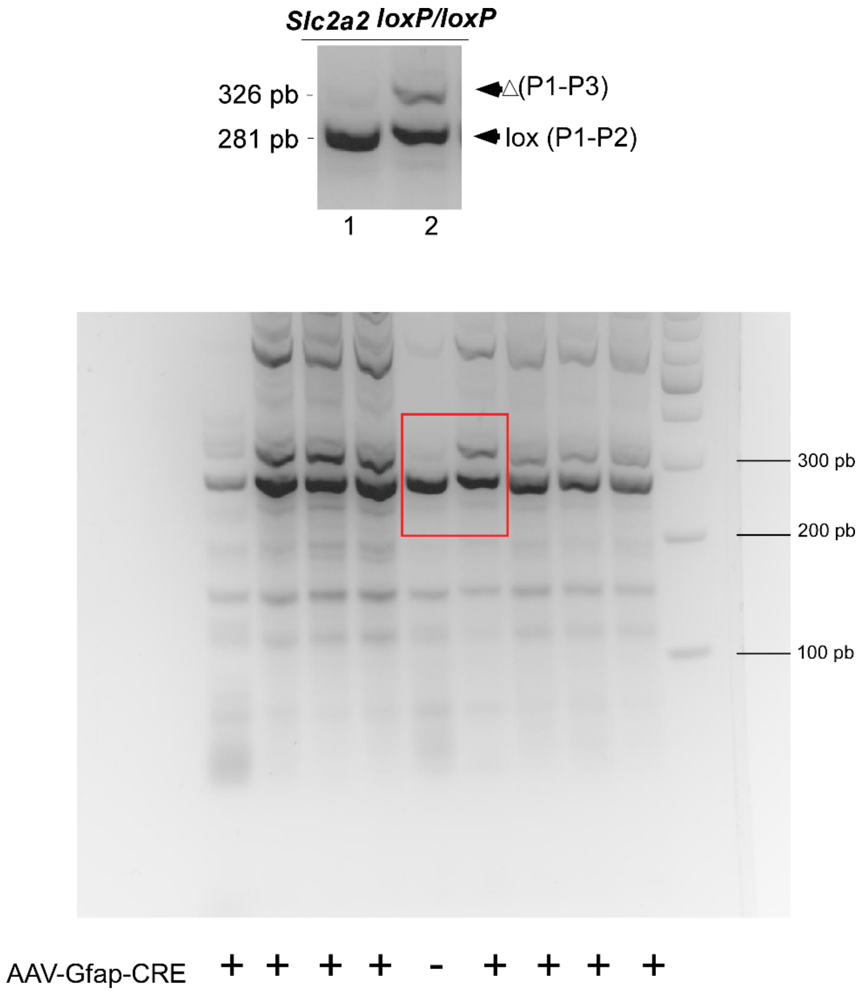

Supplementary Figure 3: Uncropped version of genomic PCR presented in the figure 3B.

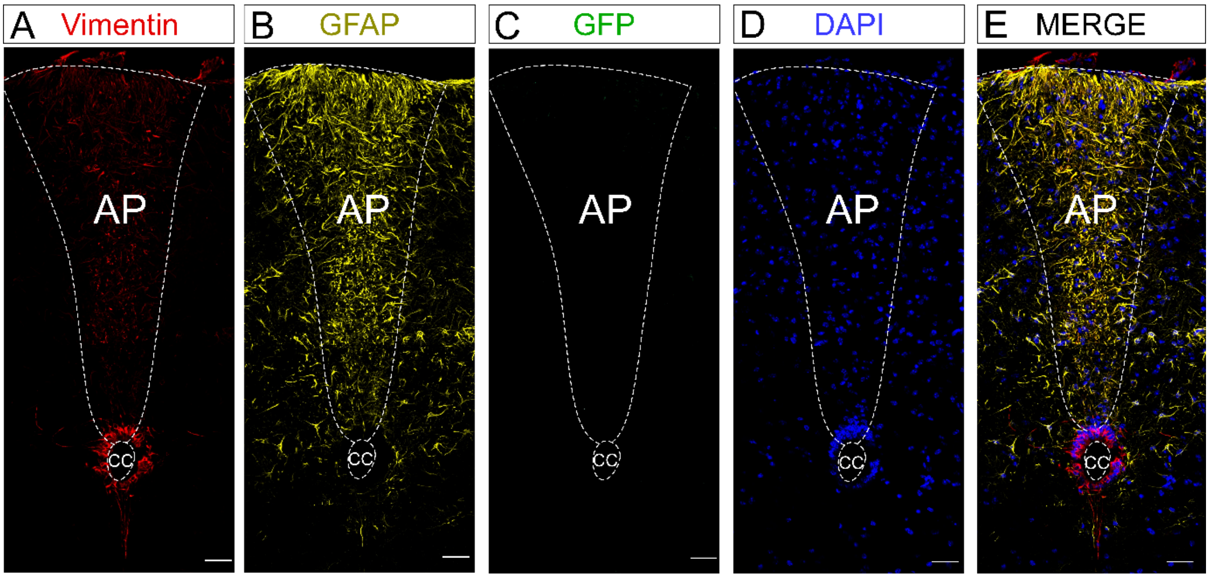

Supplementary Figure 4: AAV-*Gfap*-Cre-GFP does not transduce brainstem ependymal glial cells.

(A-E) Coronal sections at bregma AP -7.47 mm of the AP and CC (20 µm) were analyzed using the anti-vimentin (red) **(A)** and anti-GFAP (yellow) **(B)** antibodies. The GFP fluorescence (green) **(C)** was analyzed at 2-weeks post-transduction. DAPI was used as a nuclear marker **(D)**. AP: area postrema, CC: central channel. Scale bar: 50 µm.

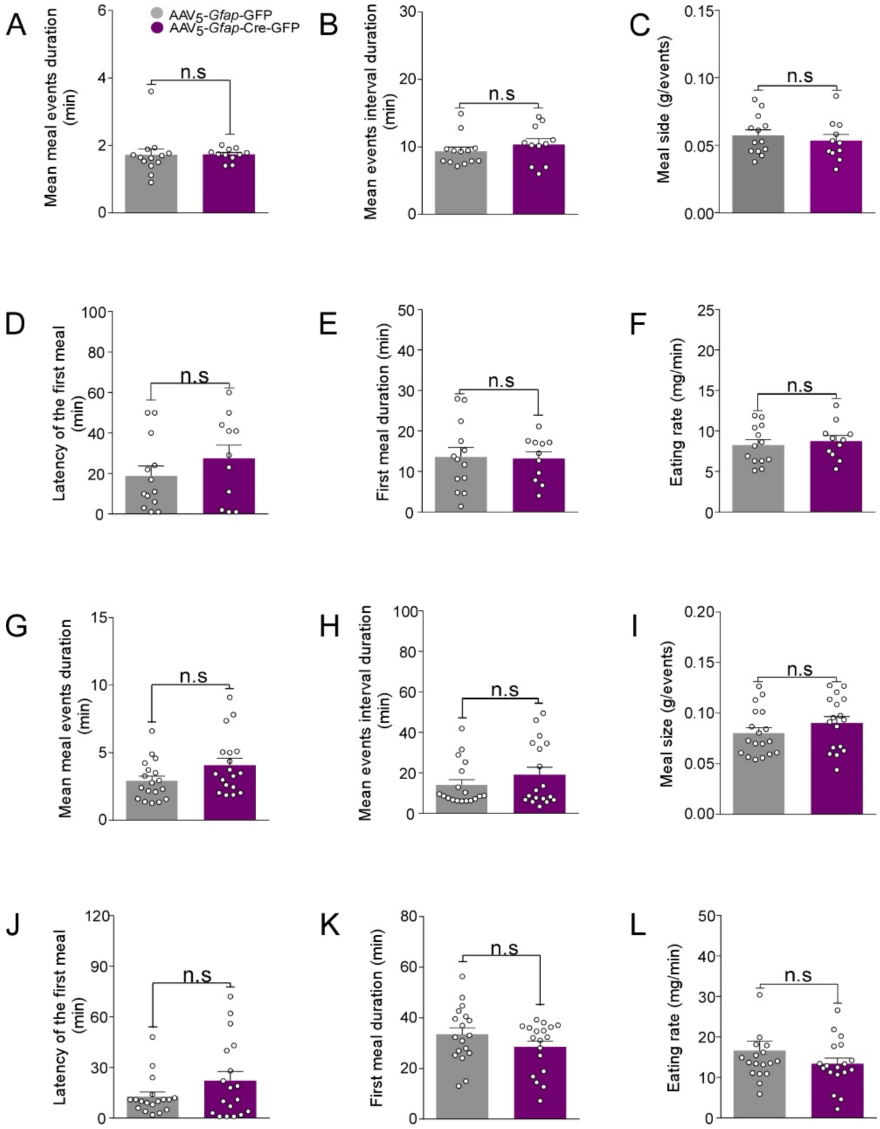

Supplementary Figure 5: *Glut2* inactivation in GFAP-expressing tanycytes does not affect satiety parameters.

(A) Mean meal events duration (min), **(B)** mean events interval duration (min), **(C)** meal side (g/events), **(D)** latency of the first meal (min), **(E)** first meal duration (min) and **(F)** eating rate (mg/min) of mice transduced for 2-weeks with the viral vector AAV-*Gfap*-GFP (gray bars, n=13-18 mice) or AAV-*Gfap*-Cre-GFP (purpure bars, n=11-18 mice) in basal condition. **(G)** Mean meal events duration (min), **(H)** mean events interval duration (min), **(I)** meal size (g/events), **(J)** latency of the first meal (min), **(K)** first meal duration (min) and **(L)** eating rate

(mg/min) during the fast-to-refeeding transition. Error bars represent SEM. Comparisons between two groups were performed using a student's *t*-test. n.s: not significant.

Supplementary Table 1: Statistical results for unpaired t, one-way and two-way ANOVA test analyses.

Figure number	Test used	n	Data reporter	P-value	t,df<br>F(DFn, DFd)	DF
**Fig. 2C**	One way ANOVA	n: 6	Mean ± SEM	P < 0,0001	F (11, 200) = 36,90	11
**Fig. 4A**	Unpaired t test	n:9	Mean ± SEM	0,6464	t=0,4676 df=16	
**Fig. 4B**	Unpaired t test	n: 11-13	Mean ± SEM	0,4799	t=0,7186 df=22	
**Fig. 4C**	Unpaired t test	n: 11-13	Mean ± SEM	0,067	t=1,927 df=22	
**Fig. 4D**	One way ANOVA	n: 11-13	Mean ± SEM	P < 0,0001	F (3, 44) = 33,22	3
**Fig. 4E**	Two Way ANOVA	n: 11-13	Mean ± SEM	Interaction: P = 0,4469<br>Row Factor: P < 0,0001<br>Colum factor: P = 0,1232	Interaction F (11, 264) = 0,9997<br>Row Factor F (11, 264) = 9,004<br>Column factor F (1, 264) = 2,391	11<br>11<br>1
**Fig. 4G**	Unpaired t test	n: 18	Mean ± SEM	0,0273	t=2,306 df=34	
**Fig. 4H**	Unpaired t test	n: 18	Mean ± SEM	0,0194	t=2,454 df=34	
**Fig. 4I**	One way ANOVA	n: 18	Mean ± SEM	P < 0,0001	F (3, 68) = 126,4	3
**Fig. 4J**	Two Way ANOVA	n: 18	Mean ± SEM	Interaction: P = 0,0314<br>Row Factor: P < 0,0001<br>Colum factor: P = 0,2548	Interaction F (11, 408) = 1,956<br>Row factor F (11, 408) = 8,281<br>Column factor F (1, 408) = 1,300	11<br>11<br>1
**Fig. 4K**	One way ANOVA	n: 18	Mean ± SEM	P = 0,0008	F (3, 68) = 6,256	3
**Fig. 4L**	Two Way ANOVA	n:11-18	Mean ± SEM	Interaction: P = 0,0682<br>Row factor: P = 0,1054<br>Colum factor: P < 0,0001	Interaction F (1, 56) = 3,458<br>Row factor: F (1, 56) = 2,708<br>Column factor F (1, 56) = 23,00	1<br>1<br>1
**Fig. 4M**	Two Way ANOVA	n:11-18	Mean ± SEM	Interaction: P = 0,0795<br>Row factor: P = 0,6724<br>Colum factor: P = 0,0005	Interaction F (1, 56) = 3,191<br>Row factor: F (1, 56) = 0,1807<br>Column factor F (1, 56) = 13,83	1<br>1<br>1
**Fig. 4N**	Two Way ANOVA	n:11-18	Mean ± SEM	Interaction: P < 0,0001<br>Row factor:P = 0,0052<br>Colum factor: P = 0,0052	Interaction F (1, 63) = 23,44<br>Row factor: F (1, 63) = 8,369<br>Column factor F (1, 63) = 8,369	1<br>1<br>1
**Fig. 5A**	Two Way ANOVA	n: 20	Mean ± SEM	Interaction: P = 0,1804<br>Row factor: P < 0,0001<br>Colum factor: P = 0,6895	Interaction F (1, 75) = 1,828<br>Row factor: F (1, 75) = 27,27<br>Column factor: F (1, 75) = 0,1608	1<br>1<br>1
**Fig. 5B**	Two Way ANOVA	n: 11-13	Mean ± SEM	Interaction: P = 0,7505<br>Row factor: P < 0,0001<br>Colum factor: P = 0,3177	Interaction F (1, 44) = 0,1024<br>Row factor: F (1, 44) = 27,48<br>Column factor F (1, 44) = 1,022	1<br>1<br>1
**Fig. 5C**	Two Way ANOVA	n: 11-12	Mean ± SEM	Interaction: P = 0,9013<br>Row factor: P = 0,0496<br>Colum factor: P = 0,0169	Interaction F (1, 43) = 0,01555<br>Row factor: F (1, 43) = 4,081<br>Column factor F (1, 43) = 6,178	1<br>1<br>1
**Fig. 5D**	Two Way ANOVA	n: 11-12	Mean ± SEM	Interaction: P = 0,5643<br>Row factor: P < 0,0001<br>Colum factor: P = P = 0,0251	Interaction F F (4, 50) = 0,7475<br>Row factor: F (4, 50) = 29,72<br>Column factor F(1, 50) = 5,336	4<br>4<br>1
**Fig. 5E**	Two Way ANOVA	n: 9-12	Mean ± SEM	Interaction: P = 0,4633<br>Row factor: P < 0,0001<br>Colum factor: P = 0,3740	Interaction F (1, 38) = 0,5489<br>Row factor: F (1, 38) = 220,8<br>Column factor F (1, 38) = 0,8094	1<br>1<br>1
**Fig. 5F**	Two Way ANOVA	n: 6-9	Mean ± SEM	Interaction: P = 0,1220<br>Row factor: P = 0,5986<br>Colum factor: P = 0,7279	Interaction F (1, 26) = 2,555<br>Row factor: F (1, 26) = 0,2840<br>Column factor F (1, 26) = 0,1237	1<br>1<br>1
**Fig. 5G**	Two Way ANOVA	n: 11-16	Mean ± SEM	Interaction: P = 0,0802<br>Row factor: P < 0,0001<br>Colum factor: P = 0,1525	Interaction F (1, 50) = 3,188<br>Row factor: F (1, 50) = 94,79<br>Column factor F (1, 50) = 2,111	1<br>1<br>1
**Fig. 5H**	Two Way ANOVA	n: 19-28	Mean ± SEM	Interaction: P = 0,8206<br>Row factor: P < 0,0001<br>Colum factor: P = 0,1027	Interaction F (1, 93) = 0,05170<br>Row factor: F (1, 93) = 27,34<br>Column factor F (1, 93) = 2,717	1<br>1<br>1
**Fig. 6A**	One way ANOVA	n: 3-4	Mean ± SEM	P = 0,0003	F (5, 15) = 9,489	5
**Fig. 6B**	One way ANOVA	n: 3-5	Mean ± SEM	P = 0,0006	F (5, 18) = 7,559	5
**Fig. 6C**	One way ANOVA	n: 3-5	Mean ± SEM	P < 0,0001	F (5, 18) = 14,45	5
**Fig. 6D**	One way ANOVA	n: 3-5	Mean ± SEM	P = 0,7607	F (5, 18) = 0,5162	5
**Fig. 6E**	One way ANOVA<br>One way ANOVA	n: 3-5<br>n: 3-4	Mean ± SEM<br>Mean ± SEM	P = 0,8264<br>P = 0,9883	F (3, 12) = 0,2977<br>F (3, 10) = 0,04086	3<br>3
**Fig. 7B**	Two Way ANOVA	n: 6-9	Mean ± SEM	Interaction: P = 0,5669<br>Row Factor: P = 0,8769<br>Column Factor: P = 0,0022	Interaction: F (1, 24) = 0,3371<br>Row Factor: F (1, 24) = 0,02449<br>Column Factor: F (1, 24) = 11,74	1<br>1<br>1
**Fig. 7C**	Two Way ANOVA	n: 7-9	Mean ± SEM	Interaction: P = 0,1892<br>Row Factor: P = 0,6839<br>Column Factor: P < 0,0001	Interaction: F (1, 25) = 1,822<br>Row Factor: F (1, 25) = 0,1697<br>Column Factor: F (1, 25) = 40,46	1<br>1<br>1
**Fig. 7D**	Two Way ANOVA	n: 6-7	Mean ± SEM	Interaction: P = 0,8050<br>Row Factor: P = 0,5009<br>Column Factor: P = 0,0093	Interaction: F (1, 23) = 0,06239<br>Row Factor: F (1, 23) = 0,4676<br>Column Factor: F (1, 23) = 8,062	1<br>1<br>1
**Sup. Fig. 5A**	Unpaired t test	n: 11-13	Mean ± SEM	P= 0,9380	t=0,07862 df=22	
**Sup. Fig. 5B**	Unpaired t test	n: 11-13	Mean ± SEM	P=0,3402	t=0,9750 df=22	
**Sup. Fig. 5C**	Unpaired t test	n: 11-13	Mean ± SEM	P=0,5185	t=0,6562 df=22	
**Sup. Fig. 5D**	Unpaired t test	n: 11-13	Mean ± SEM	P=0,2811	t=1,105 df=22	
**Sup. Fig. 5E**	Unpaired t test	n: 11-13	Mean ± SEM	P=0,8978	t=0,1300 df=22	
**Sup. Fig. 5F**	Unpaired t test	n: 11-13	Mean ± SEM	P=0,6123	t=0,5141 df=22	
**Sup. Fig. 5G**	Unpaired t test	n: 18	Mean ± SEM	P=0,0665	t=1,896 df=34	
**Sup. Fig. 5H**	Unpaired t test	n: 18	Mean ± SEM	P=0,2733	t=1,114 df=34	
**Sup. Fig. 5I**	Unpaired t test	n: 18	Mean ± SEM	P=0,2407	t=1,195 df=33	
**Sup. Fig. 5J**	Unpaired t test	n: 18	Mean ± SEM	P=0,1268	t=1,565 df=34	
**Sup.Fig. 5K**	Unpaired t test	n: 18	Mean ± SEM	P=0,1667	t=1,413 df=34	
**Sup.Fig. 5L**	Unpaired t test	n: 18	Mean ± SEM	P=0,2471	t=1,178 df=34	

Table showing the number of animals used in each experiment, test used, data reporter, p-value, statistical values and degrees of freedom. **n**: Number of animals used for the experiment; **SEM**, standard error of the mean; ***t***, unpaired t-test statistic; **DF**, degrees of freedom; **DFn**, numerator degrees of freedom; **DFd**, denominator degrees of freedom. Two groups comparison was performed using an unpaired *t*-test. The comparison between more of one non-parametric group was performed using a One-way ANOVA. Comparisons between two groups and two variables were made by applying a Two way-ANOVA.
